# Optimal parameters in variable‐velocity scanning luminescence lifetime microscopy

**DOI:** 10.1002/jemt.23566

**Published:** 2020-07-28

**Authors:** Zdeněk Petrášek, Juan M. Bolivar, Bernd Nidetzky

**Affiliations:** ^1^ Institute of Biotechnology and Biochemical Engineering Graz University of Technology, NAWI Graz Graz Austria; ^2^ Austrian Centre of Industrial Biotechnology Graz Austria; ^3^ Present address: Chemical and Materials Engineering Department Complutense University of Madrid Madrid Spain

**Keywords:** confocal laser scanning microscopy, error analysis, lifetime imaging, luminescence, phosphorescence

## Abstract

We determine the optimal parameters (scan velocities) for measuring the luminescence lifetime on the microsecond scale using the recently introduced method based on scanning the excitation beam across the sample. Using simulations, we evaluate the standard deviation and bias of the luminescence decay rate determined by scanning with two different velocities. The analysis is performed for Poisson‐ and normal‐distributed signals, representing different types of detection techniques. We also show that a weak uncorrected background induces a bias in the obtained decay rate, and take this effect into account when choosing optimal measurement parameters. For comparison, the analysis is additionally performed for two conventional gating schemes for lifetime measurement. The variable‐velocity scanning method is found to be more robust to the effect of the background signal than the gating schemes.

## Graphical abstract

- The scan velocities that minimize the error of lifetime determination in variable‐velocity scanning luminescence lifetime imaging have been identified.
- The variable‐velocity scanning method is found to be more robust to the effect of the background signal than the conventional gating schemes.

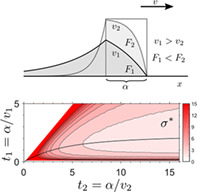

## 1 INTRODUCTION

The luminescence lifetime is an important parameter often employed for sensing the environment and the interactions of the emitting molecules, or simply for distinguishing between dyes of similar spectral properties (Lakowicz, 2006; Meyer‐Almes, 2017; Suhling, French, & Phillips, 2005; Valeur, 2001). The independence of lifetime on the excitation intensity and on the dye concentration within a broad experimental range is a major advantage over measurements based only on emission intensity, and a reason for the many applications of time‐resolved fluorescence (Bolivar, Consolati, Mayr, & Nidetzky, 2013; Gruber, Marques, Szita, & Mayr, 2017; Wang & Wolfbeis, 2014). However, the experimental determination of luminescence lifetime typically requires complex equipment, involving pulsed excitation and time‐resolved detection. Furthermore, analytics based on lifetime generally necessitates a higher signal‐to‐noise ratio than methods using solely emission intensity. This is especially relevant in microscopy applications, where the single volumes within which the measurements are performed (pixels, voxels) are small, and the signals are limited. The particular choice of experimental parameters can, therefore, have profound effects on the quality of the results, quantified by the standard deviation and bias of the determined lifetime or decay rate.

For these reasons, the question of the optimal choice of parameters in different methods of lifetime measurement has been addressed in the past (Ballew & Demas, 1989, Ballew & Demas, 1991; Hall & Selinger, 1981; Good, Kallir, & Wild, 1984; Heeg, 2013, 2014; Köllner & Wolfrum, 1992; Moore, Chan, Demas, & DeGraff, 2004; Peng, Liu, Zhao, & Kim, 2016; Santra et al., 2016; Tellinghuisen & Wilkerson, 1993; Xu, Qiao, Nie, & Zhang, 2016). Theoretical studies of time‐domain methods, where the sample is excited by a short pulse and the decay subsequently detected in a variable number of time channels (gates), have focused on various aspects of the detection scheme: the number of time channels and their widths (Hall & Selinger, 1981; Köllner & Wolfrum, 1992; Moore et al., 2004), the channel overlap (Chan, Fuller, Demas, & DeGraff, 2001; Heeg, 2014; Moore et al., 2004), the effect of different noise distributions (Heeg, 2013, 2014; Tellinghuisen & Wilkerson, 1993), the effect of background (Ballew & Demas, 1991; Köllner & Wolfrum, 1992; Moore et al., 2004; Soper & Legendre, 1994), etc., and have identified the optimal parameters and quantified the expected errors.

We have recently introduced an alternative method for the measurement of luminescence lifetimes on the microsecond scale based on scanning the beam across the sample with different velocities and simultaneously detecting the emitted signal (Petrášek, Bolivar, & Nidetzky, 2016). The technique does not require pulsed excitation and relies on the excitation‐time dependence on the scan velocity. Different scan velocities result in different luminescence signals, and measurements at as few as two velocities are sufficient to determine the luminescence lifetime. The precision of the lifetime measured in this way depends on the choice of the two scan velocities. As the principle of the variable‐velocity scanning method differs from the conventional time‐gating approach, the existing studies dealing with optimal parameter choice do not apply here. In this work, we used simulations and analytical calculations to determine the optimal scan speeds that minimize the standard deviation of the decay rate for the measurement scheme employing two velocities. We take into account the effect of small uncorrected background that can bias the resulting decay rate. For comparison, the analysis is performed also for two conventional gating schemes with generally unequal time gates, one with non‐overlapping consecutive gates and the other one with fully overlapping gates. The presented analysis and results are going to facilitate the implementation and application of the variable‐velocity scanning method, particularly in combination with confocal laser scanning microscopy.

## 2 THEORY

### 2.1 Lifetime measurement with the variable‐velocity scanning method

**FIGURE 1:**
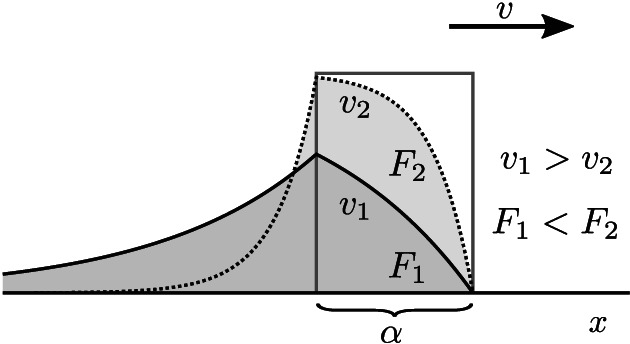
The principle of the variable‐velocity scanning method: the detection area of the size *α* where the molecules are excited moves across the sample (dimension *x*). The detected signal *F*_*i*_ (integrated over the area *α*) depends on the scan velocity *v*_*i*_; this dependence is used to determine the luminescence lifetime. The vertical axis represents the emission intensity

The measurement of the luminescence lifetime by employing different scan velocities takes advantage of the finite time needed to populate the excited state in an ensemble of molecules from the start of the excitation (Petrášek et al., 2016). This time is determined predominantly by the luminescence decay rate. The scan velocity of the excitation beam determines the duration of excitation at any point within the sample. At fast scan speeds, resulting in an excitation period that is short compared to the luminescence lifetime, the excited state will not have reached its steady‐state population and the emitted signal will be relatively low. At slower scan speeds, where the population of the excited state gets closer to the steady‐state, the emitted signal will be on average higher (Figure 1). The luminescence lifetime can be determined from the intensities measured at least two different scan speeds.

We have previously shown that the dependence on the scan velocity *v* of the luminescence intensity, *f*, can be expressed as (Petrášek et al., 2016):

(1) $$f\left(v\right)=f_{0}\left(1-\frac{v}{\mathrm{\alpha k}}\left(1-e^{-\mathrm{\alpha k}/v}\right)\right),$$

where *k* is the decay rate (the inverse of the luminescence lifetime *τ*: *τ* = 1/*k*), and *α* is a factor nominally equal to the linear size of the illuminated area, typically a focused laser beam in a confocal laser scanning microscope. In practice, *α* is determined by calibration with a dye of a known luminescence lifetime. The emission intensity increases with decreasing scan velocity, and in the limit of very low velocity approaches the steady‐state value *f*_0_.

**FIGURE 2:**
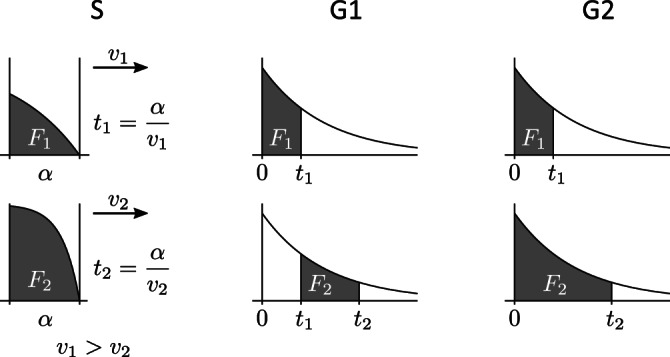
The three measurement schemes analyzed in this work. S: the scanning method employing two different scan velocities; G1, G2: time‐gating methods using pulsed excitation at time *t* = 0 and detection in two‐time windows. In the Scheme G1, the second time window begins immediately after the first one (no overlap); in the Scheme G2 both windows start at the time *t* = 0 (full overlap)

When scanning with velocity *v*, any position within the sample is illuminated for time *t* = *α*/*v*. Employing two different scan speeds gives two illumination times *t*_1_ and *t*_2_. In the Results section, we determine the optimal times *t*_1_ and *t*_2_, from which the scan velocities *v*_1_ and *v*_2_ can be calculated (Figure 2).

In order to obtain the signals *F*_1_ and *F*_2_, the luminescence intensity is integrated for times *t*_1_′ and *t*_2_′. In a confocal laser scanning microscope, these times correspond to the time per pixel *t*_*i*_ ′  = *d*/*v*_*i*_, where *d* is the pixel size. It follows from Equation (1) (substituting *t*_*i*_ = *α*/*v*_*i*_):

(2) $$F_{1}=f\left(v_{1}\right)t_{1}\prime =f_{0}\left(1-\frac{1-e^{-kt_{1}}}{kt_{1}}\right)t_{1}\prime ,\;\;F_{2}=f\left(v_{2}\right)t_{2}\prime =f_{0}\left(1-\frac{1-e^{-kt_{2}}}{kt_{2}}\right)t_{2}\prime .$$

The integration times *t*_1_′ and *t*_2_′ are generally different from the illumination times *t*_1_ and *t*_2_, in the same way as the size *d* of an image pixel is usually not the same as the size *α* of the focused laser spot in the sample. Substituting *t*_*i*_ ′  = (*d*/*α*)*t*_*i*_ in Equation (2) introduces a constant factor *d*/*α*, which, however, cancels out in the calculation of *k* because only the ratios of intensities *F*_*i*_ are used, as explained in detail below. Therefore, without a loss of generality, we will assume in the following that these times are the same: *t*_1_ ′  = *t*_1_, *t*_2_ ′  = *t*_2_. The two equations above (Equation (2)) can then be used to calculate the decay rate *k* from the measured signals *F*_1_ and *F*_2_ and the known times *t*_1_ and *t*_2_. In the following text, this measurement method will be denoted as the Scheme S (“scanning”).

### 2.2 Conventional gating schemes

We compare the scanning method (Scheme S) with the commonly employed time‐gating method, which relies on pulsed excitation and time‐resolved detection of the subsequent light emission in several time windows (Suhling et al., 2005; Webb et al., 2002).

Two gating schemes are considered here, each with two‐time channels (Figure 2). In both cases, the first gate starts at time zero, immediately after the pulsed excitation. In the scheme denoted G1 ('gating') the second gate starts immediately after the end of the first gate; the gates generally have different widths: *t*_1_ and *t*_2_ − *t*_1_. In the second scheme, denoted G2, the second gate also starts at time zero, so both gates are fully overlapped, and the unequal gate widths in this case are *t*_1_ and *t*_2_.

Assuming a mono‐exponential luminescence decay *f*(*t*):

(3) $$f\left(t\right)=f_{0}e^{-\mathrm{kt}},$$

the intensities *F*_1_ and *F*_2_ detected in the two‐time windows in the G1 scheme are:

(4) $$F_{1}=\frac{f_{0}}{k}\left(1-e^{-kt_{1}}\right),\;F_{2}=\frac{f_{0}}{k}\left(e^{-kt_{1}}-e^{-kt_{2}}\right),$$

and in the G2 scheme:

(5) $$F_{1}=\frac{f_{0}}{k}\left(1-e^{-kt_{1}}\right),\;\;F_{2}=\frac{f_{0}}{k}\left(1-e^{-kt_{2}}\right).$$

### 2.3 Calculation of the decay rate ***k***

The experimentally measured or simulated signals in the two‐time windows in any of the three measurement schemes are denoted *N*_1_ and *N*_2_. These values are subject to experimental noise, and therefore in practice differ from the theoretical values *F*_1_ and *F*_2_. The signals *N*_1_ and *N*_2_ may be photon counts, in case of photon‐counting detection typically used with weak signals (confocal microscope), or real numbers resulting from an analog‐to‐digital conversion of an analog detector signal. In this study, we consider two possible statistical distributions of the values *N*_1_ and *N*_2_: Poisson and normal (Gaussian), both with their means equal to *F*_1_ and *F*_2_. While the Poisson distribution describes photon‐counting detection, we use the normal distribution with the variance proportional to the detection time window Δ*t* (Figure 2) to approximate detection with additional sources of noise, such as noise related to the background.

For both distributions the maximum‐likelihood criterion (Bajzer, Therneau, Sharp, & Prendergast, 1991; Tellinghuisen & Wilkerson, 1993) for parameter estimation leads to the following equations, from which the unknown parameters *f*_0_ and *k* can be determined:

(6) $$N_{1}=F_{1},\;\;N_{2}=F_{2}.$$

Since we are interested only in the decay rate *k*, it is practical to calculate the following ratio *r* from the experimental data:

(7) $$r=\frac{N_{1}}{N_{1}+N_{2}}.$$

Using Equation (6) for all three measurement schemes, we obtain the equation, from which the decay rate *k* can be calculated numerically: the Scheme S:

(8) $$r={\left(1+\frac{1-e^{-kt_{2}}-kt_{2}}{1-e^{-kt_{1}}-kt_{1}}\right)}^{-1},$$

the Scheme G1:

(9) $$r=\frac{1-e^{-kt_{1}}}{1-e^{-kt_{2}}},$$

and the Scheme G2:

(10) $$r={\left(1+\frac{1-e^{-kt_{2}}}{1-e^{-kt_{1}}}\right)}^{-1}.$$

The signal strength in the three measurement schemes is expressed by the parameter *f*_0_ (Equations (1) and (3)). It is convenient to substitute *f*_0_ = *nk* in the equations defining *F*_1_ and *F*_2_ (Equations (2), (4), and (5)), where the new parameter *n* replaces *f*_0_, and can be interpreted as the number of photon counts if photon‐counting detection is used, or as a time‐integrated signal in general. In Scheme S, the parameter *n* represents the signal detected by integration over the time *t* ′  = 1/*k* equal to the luminescence lifetime in the steady‐state regime (*kt*_*i*_ ≫ 1, Equation (2)). In the Schemes G1 and G2, *n* is the total signal detected after one excitation pulse.

The numerical values of the times *t*_1_ and *t*_2_ in the simulation results, in figures, and in‐text are given in relative units of 1/*k*.

### 2.4 The effect of the background

The presence of a constant background signal influences the measured decay rate *k*. If the background is not taken into account during the analysis, it biases the calculated value of *k*. Even if included in the fitting model as an unknown parameter, the background affects the precision with which *k* is determined, and also influences the optimal experimental parameters (*t*_1_, *t*_2_). It has been shown that even if the correct background is subtracted, the noise associated with the background negatively influences the precision with which the decay rate can be determined (Heeg, 2013; Köllner & Wolfrum, 1992).

The background effect has been described for the gating Schemes (G1) several times in the past (Ballew & Demas, 1991; Heeg, 2013, 2014; Köllner & Wolfrum, 1992; Moore et al., 2004). The best practice is to determine the background independently, and include it in the analysis as a known parameter. This is, however, not always possible or cannot be done exactly. Consequently, a small contribution of the background still remains in the signal. Here, we analyze the bias in *k* when the background is not corrected for, or when the residual background remains, and show that the impact of background differs widely among the considered measurement schemes. Even at low background levels, its effect has to be taken into account when choosing *t*_1_ and *t*_2_.

The background intensity *B* is assumed to be constant in time, and we express it relatively to the luminescence amplitude *f*_0_ in Equations (1) and (3): *B* = *bf*_0_. If *b* = 1, the background is comparable to the luminescence signal; we are, however, interested in the case of low background: *b* < 1. The background intensity *B* multiplied by the corresponding gate time width then represents the contribution of background to the signals *F*_*i*_. Adding the background to the signals *F*_1_ and *F*_2_ results in background‐affected signals *F*_1_′ and *F*_2_′ and their ratio *r*′:

(11) $$F_{1}\prime =F_{1}+Bt_{1},\;F_{2}\prime =F_{2}+Bt_{2},$$

(12) $$r^{\prime }={\left(1+\frac{1-e^{-kt_{2}}-k\left(1+b\right)t_{2}}{1-e^{-kt_{1}}-k\left(1+b\right)t_{1}}\right)}^{-1}$$

for the Scheme S (where *F*_1_ and *F*_2_ are taken from Equations (2)), and:

(13) $$F_{1}\prime =F_{1}+Bt_{1},\;F_{2}\prime =F_{2}+B\left(t_{2}-t_{1}\right),$$

(14) $$r\prime =\frac{1-e^{-kt_{1}}+\mathrm{bk}t_{1}}{1-e^{-kt_{2}}+\mathrm{bk}t_{2}}$$

for the Scheme G1 (where *F*_1_ and *F*_2_ are taken from Equations (4)). The bias of *k* as a result of background in the measurement Scheme G2 is the same as in the Scheme G1, therefore, it is not shown it explicitly here.

In order to calculate the biased decay rate *k*′, the ratio *r*′ is calculated from the equations above (Equations (12) and (14)), substituted to the equation for unbiased *r* (*r* = *r*′) without any background (Equations (8) and (9)), and subsequently the biased *k*′ is calculated from *r*.

## 3 METHODS

For all the measurement schemes (Figure 2) the noisy experimental signals *N*_1_ and *N*_2_ were simulated (Monte Carlo) and analyzed to obtain the decay rate *k*, in the following way. The signals *N*_1_ and *N*_2_ were generated as random numbers with Poisson or normal (Gaussian) distribution, with the mean given by *F*_1_ and *F*_2_ in Equations (2), (4), and (5), and the variance (normal distribution) equal to *nΔt*, where Δ*t* is the width of the time window (Δ*t* = *t*_1_, Δ*t* = *t*_2_ or Δ*t* = *t*_2_ − *t*_1_ depending on the measurement scheme; see Figure 2). Then, the ratio *r* (Equation (7)) was calculated from *N*_1_ and *N*_2_, and subsequently the estimate of the decay rate *k* was calculated from *r* by numerically inverting Equations (8), (9), or (10), depending on the measurement scheme.

The simulations were performed for ten different signal levels *n*, ranging from 100 to 10^5^. The simulation was repeated 10,000 times for every set of parameters, the obtained rates *k* were averaged and their standard deviation *σ*_*k*_ and bias relative to the true value *k*_0_ were calculated. All simulations were done in Matlab (The MathWorks, Natick, MA).

The standard deviation *σ*_*k*_ of the decay rate *k* was also estimated analytically using the standard error propagation method. The *σ*_*k*_ is related to the standard deviation $\sigma_{F_{i}}$ of the signals *F*_*i*_ as follows:

(15) $$\sigma_{k}^{2}={\left(\frac{\partial k}{\partial F_{1}}\right)}^{2}\sigma_{F_{1}}^{2}+{\left(\frac{\partial k}{\partial F_{2}}\right)}^{2}\sigma_{F_{2}}^{2}={\left(\frac{\partial k}{\partial r}\right)}^{2}\left[{\left(\frac{\partial r}{\partial F_{1}}\right)}^{2}\sigma_{F_{1}}^{2}+{\left(\frac{\partial r}{\partial F_{2}}\right)}^{2}\sigma_{F_{2}}^{2}\right]$$

The derivatives in Equation (15) were determined from Equations (6), (7), (8), (9), (10)). The analytical calculations of error propagation were done in Mathematica (Wolfram Research Inc., Champaign, IL).

## 4 RESULTS AND DISCUSSION

### 4.1 The standard deviation of the decay rate

In order to determine the optimal time windows *t*_1_ and *t*_2_ for all three measurement schemes, we simulated the measurement signals *N*_1_ and *N*_2_ for a range of values of *t*_1_ and *t*_2_ and calculated the decay rate *k* using Equations (8), (9), (10)). The presence of noise, the level of which is determined by the parameter *n*, means that the calculated rate *k* was distributed around the true value *k*_0_. By repeating the simulations many times, we could evaluate the mean value of *k*, its bias from the true value *k*_0_, and the standard deviation *σ*_*k*_.

**FIGURE 3:**
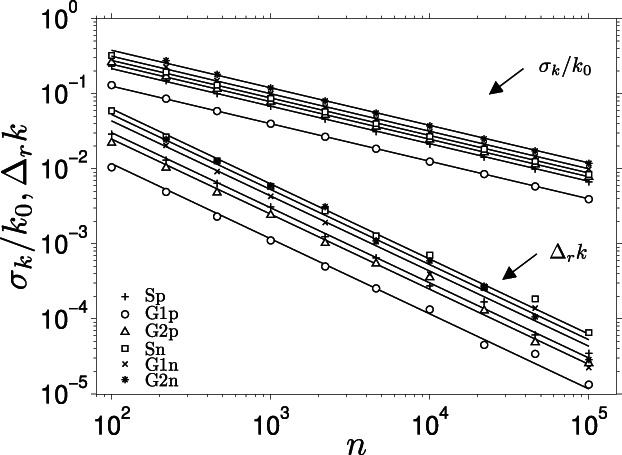
The dependence of the relative standard deviation *σ*_*k*_/*k*_0_ and the relative bias Δ_*r*_*k* on the signal strength *n* as determined from simulations. The plotted values are taken at *t*_1_ and *t*_2_, for which the minimum of *σ*_*k*_ was found within the tested range (Figure 4). The results are shown for the three measurement Schemes (S, G1, and G2) with either Poisson (p) or normal‐distributed (n) signal

The standard deviation *σ*_*k*_ of the decay rate *k* depends on the number of counts *n* as follows (Figure 3):

(16) $$\frac{\sigma_{k}}{k_{0}}=\frac{\sigma^{*}}{\sqrt{n}}$$

where the parameter σ^*^ differs among the considered measurement schemes, and is a measure of the precision of the method. The signal strength *n* was found to have no influence on σ^*^, meaning that the optimal parameters determined below are independent of the signal intensity.

**FIGURE 4:**
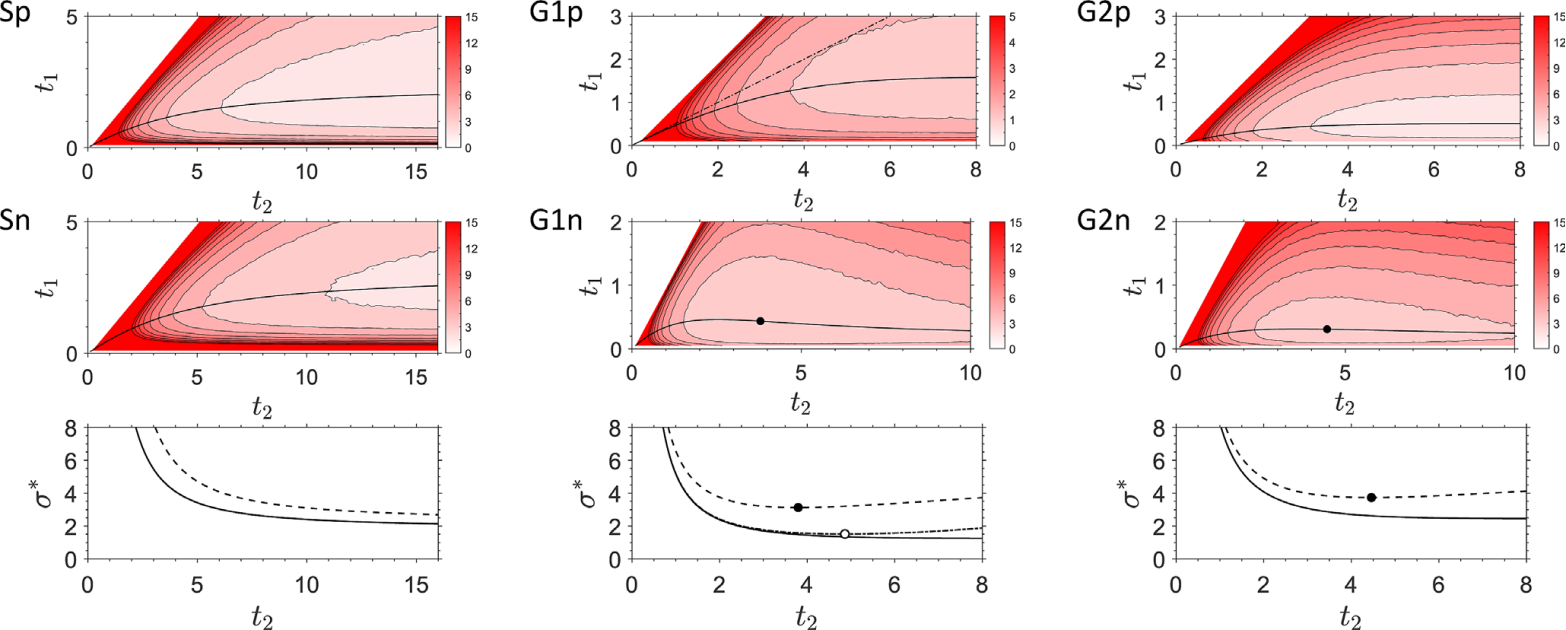
The dependence of the parameter σ^*^, describing the relative standard deviation *σ*_*k*_/*k*_0_ (Equation (16)) of the decay rate *k*, on the times *t*_1_ and *t*_2_. The three columns correspond to the measurement Schemes S (left), G1 (middle), and G2 (right). The data in the first row were Poisson‐distributed, in the second row normal‐distributed. The solid lines indicate the minimum of σ^*^ for a given value of *t*_2_. If an absolute minimum is present, it is indicated by a black dot. The bottom row shows the minimum values of σ^*^ for a given *t*_2_ (solid lines: Poisson‐distributed data, dashed lines: normal‐distributed data). The dash‐dotted line in Scheme G1 Poisson (middle top) describes the situation when the two gates are equal (*t*_2_ = 2*t*_1_); the minimum of σ^*^ in this case is indicated by a dot‐dashed line and an empty symbol in the bottom middle graph. The times *t*_1_ and *t*_2_ are given in relative units of 1/*k* [Color figure can be viewed at wileyonlinelibrary.com]

The position of the minimum of σ^*^ indicates the optimal times *t*_1_ and *t*_2_ (Figure 4). The value of σ^*^ for the scanning method (Scheme S) decreases steadily with increasing time *t*_2_, however, from *t*_2_ ∼ 8, this decrease is not significant. The optimal time *t*_1_ then lies between 2 and 2.5 for the Poisson noise, depending on the particular value of *t*_2_, but with a rather weak sensitivity to its exact value. The situation is very similar in the case of the Gaussian noise, with the optimal value of *t*_1_ between 2 and 3.2.

Similarly, in the two gating Schemes G1 and G2 with Poisson noise the relative standard deviation σ^*^ steadily decreases with *t*_2_, with little significant decrease above *t*_2_ = 4. The optimal value of *t*_1_ is different for each scheme: it lies between 1.3 and 1.6 for the G1 scheme, and is approximately equal to 0.5 for the G2 scheme.

Contrary to the other cases, the gating Schemes G1 and G2 with normally‐distributed signals exhibit a well‐defined minimum of σ^*^: at *t*_2_ = 3.80, *t*_1_ = 0.44 for the G1 scheme, and at *t*_2_ = 4.46, *t*_1_ = 0.31 for the G2 scheme. The existence of the minimum can be understood by realizing that extending the gate width beyond the times when the luminescence has decayed only adds more noise but no signal, thus decreasing the signal‐to‐noise ratio.

For comparison with previously published results, we also looked for the optimum gate width in the Scheme G1 in the situation where the widths of both gates are equal (*t*_2_ = 2*t*_1_). This is equivalent to finding the minimum of σ^*^ along the line *t*_2_ = 2*t*_1_ in the corresponding plot in Figure 4 (dash‐dotted line). The optimal gate width in this situation is *t*_1_ = 2.43, in agreement with previous results (Ballew & Demas, 1989; Chan et al., 2001; Köllner & Wolfrum, 1992).

The standard deviation obtained from simulations (Figure 4) agrees with analytical calculations using standard error propagation method. The minimum of σ^*^ (Equation (16)) found in simulations coincides with (Schemes G1, G2) or is slightly larger (Scheme S) than the value found analytically. The reason for the small difference is the limited range of *t*_1_ and *t*_2_ explored in the simulations. Using analytical calculations we could find the asymptotic values for *t*_2_ → ∞. The positions of the minima and the analytical values of σ^*^ at the minima are summarized in Table 1.

**TABLE 1:** The positions and values of the minima of normalized standard deviation and bias of the decay rate *k* expressed by σ^*^ (Equation (16)) and *γ* (Equation (18)) for several measurement schemes and noise distributions (p: Poisson, n: normal)

Scheme	*t*_1_	*t*_2_	σ^*^	*γ*
G1p	1.59	∞	1.24	1.17
G2p	0.51	∞	2.44	2.48
Sp	2.48	∞	1.77	3.02
G1n	0.44	3.80	3.13	4.33
G2n	0.31	4.46	3.73	5.28
Sn	3.21	∞	2.16	6.27

In addition to the standard deviation, the simulations allowed us to evaluate the bias of the calculated decay rate *k*. For this purpose, we define the relative bias Δ_*r*_*k* as a difference between the calculated (*k*) and true (*k*_0_) decay rate relative to the true decay rate:

(17) $$\Delta_{r}k\equiv \left(k-k_{0}\right)/k_{0}$$

The bias was found to decrease with the number of counts *n* in the following way (Figure 3):

(18) $$\Delta_{r}k=\frac{\gamma }{n}$$

The bias parameter *γ* (Equation (18)) was determined from simulations with a range of *n* at the position of the minimum of standard deviation within the range of tested times *t*_1_ and *t*_2_, and is listed in Table 1.

The bias turns out to be positive and rather small compared to the expected error expressed by the standard deviation (Figure 3). Even at the smallest considered signal *n* (*n* = 100), where the bias is strongest, it is about one order of magnitude smaller than the standard deviation. This means that this bias can be usually ignored, or, at the weakest signal levels on the order of 100 counts per decay, corrected for by using the curves in Figure 3.

Table 1 summarizes the position (*t*_1_ and *t*_2_) of the minimum of the standard deviation σ^*^ for the considered measurement schemes, together with the standard deviation (σ^*^) and bias (*γ*) parameters at this minimum. As mentioned above, in the absence of a localized minimum for finite *t*_2_, there is a value of *t*_2_, beyond which the standard deviation does not significantly decrease, and further extension of *t*_2_ does not make a practical difference. These results, however, apply to an idealized situation, and as we show in the next section, the presence of even a small background influences the choice of the optimal *t*_1_ and *t*_2_ in a more realistic setting.

The value of the parameter σ^*^ in Table 1, together with Equation (16) can be used to estimate the signal needed to reach a required measurement error. For example, if a relative error of 10% is tolerable (*σ*_*k*_/*k*_0_ = 0.1), the signal *n* detectable over the lifetime 1/*k* should be at least 313 counts per luminescence lifetime in the reaction Scheme S with Poisson noise. For an exemplary lifetime of 4 *μs* this corresponds to a detection rate of ∼7.8 × 10^7^ counts per second. Since such a detection rate is rather high, the same signal level can be effectively achieved by either repeating the measurement more times (for example, 20× if the rate is ∼4 × 10^6^ counts per second), by binning neighboring pixels in an image or by a combination of both approaches. For comparison, under the same conditions the gating Scheme G1 requires a signal level of *n* = 154 counts per luminescence lifetime that is approximately one half.

### 4.2 The effect of uncorrected background

When the detected signal contains a constant background in addition to the luminescence, failure to fully account for the background in analysis leads to an additional bias in the measured luminescence decay rate.

**FIGURE 5:**
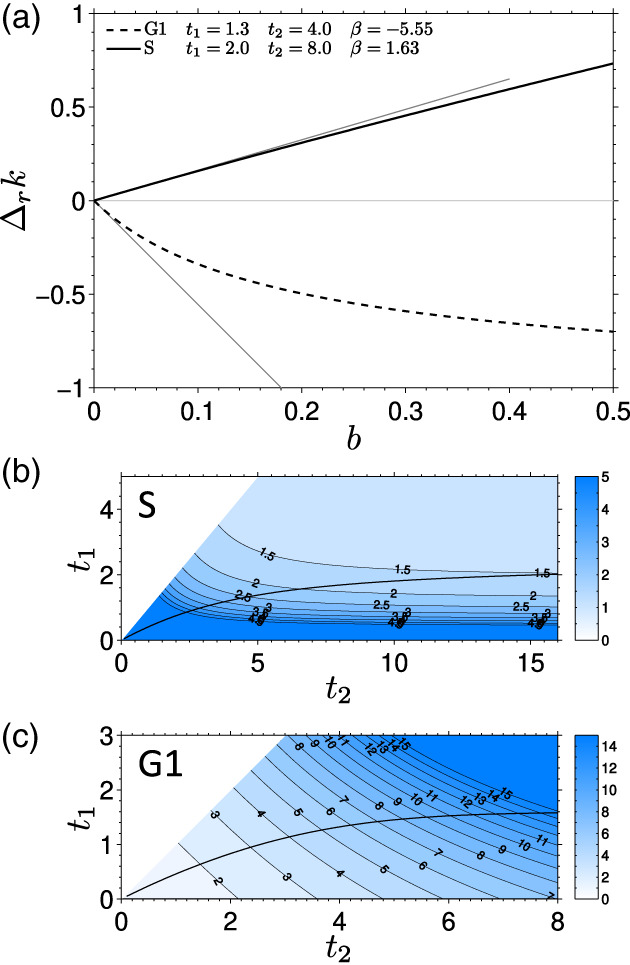
A: the bias Δ_*r*_*k* of the decay rate *k* in dependence of the background level *b* for the two measurement schemes: S (solid line) and G1 (dashed line). The bias is calculated for the following choice of the times *t*_1_ and *t*_2_: Scheme S: *t*_1_ = 2.0, *t*_2_ = 8.0; Scheme G1: *t*_1_ = 1.3, *t*_2_ = 4.0. The gray lines indicate the linear slopes in the limit of low background (*b* → 0). B, C: The dependence of the absolute value of the bias slope ∣*β*∣ (Equation (19)) on the times *t*_1_ and *t*_2_ for the Schemes S (B) and G1 (C). The solid line indicates the minimum of the standard deviation of *k* for a given value of *t*_2_ [Color figure can be viewed at wileyonlinelibrary.com]

The sensitivity of the relative bias Δ_*r*_*k* (Equation (17)) to the background depends on the chosen values of *t*_1_, *t*_2_. Figure 5a shows how the bias depends on the background level for the two measurement Schemes S and G1, with *t*_1_, *t*_2_ chosen so that the standard deviation of *k* is near its minimum for each measurement scheme. The presence of background leads to overestimated values of *k* in the measurement Scheme S (positive bias), and to lower *k* (longer lifetime) in the measurement Scheme G1 (negative bias).

Since we are interested in the effects of a weak background (small *b*), we can approximate the bias of the rate *k* for a low background *b* by a linear function with a slope *β*:

(19) $$\Delta_{r}k=\mathrm{\beta b}$$

The parameter *β* is then a dimensionless number expressing the sensitivity of *k* to the background that has not been accounted for. A comparison of *β* values (the ratio of their absolute values) of the two schemes shows that, for the *t*_1_ and *t*_2_ values chosen in Figure 5a, the gating Scheme G1 is about 3.4× more sensitive to the low background than the Scheme S.

The bias of *k*, as expressed by the sensitivity to background *β*, depends on the times *t*_1_ and *t*_2_. We have calculated *β* as explained above for the same range of *t*_1_ and *t*_2_ as the standard deviation σ^*^ (Figure 4), and show its absolute value in Figure 5b,c. These results reveal a substantial difference in the sensitivity to the background between the two considered schemes.

In the Scheme S, *β* decreases with increasing *t*_1_ and *t*_2_ (Figure 5b). This means that in order to minimize the background effects, it is favorable to choose a rather long time *t*_2_. This is the same conclusion as that reached by observing the dependence of the standard deviation σ^*^ of the decay rate on *t*_2_ (Figure 4, Table 1). For *t*_2_ larger than approximately eight, the decrease in σ^*^ and *β* is not particularly strong, therefore, any value above eight is close to optimal.

Contrary to this, in the Scheme G1, ∣*β*∣ increases with increasing *t*_1_ and *t*_2_ (Figure 5c). Comparing this with the dependence of the standard deviation σ^*^ on *t*_2_ (Figure 4) means that a compromise has to be found, where *t*_2_ is high enough to keep σ^*^ small and at the same time sufficiently low to minimize the bias due to the background. The final choice of *t*_1_ and *t*_2_ will depend on the particular circumstances of the experiment: the tolerable standard deviation of *k*, the actual expected background level, and the maximum acceptable bias due to the background.

These observations allow us to make the following suggestions for the choice of the times *t*_1_ and *t*_2_ (all expressed in relative units of 1/*k*). The optimal *t*_2_ for the Scheme S is any value larger than approximately eight, as there is no significant decrease of σ^*^ beyond this value. The optimal *t*_1_ lies between 2 and 3.2, depending on the chosen *t*_2_, and on whether the noise character is closer to the Poisson or the normal distribution (Figure 4, Table 1).

The optimal *t*_2_ for the Schemes G1 and G2 will typically be around *t*_2_ = 4, since higher *t*_2_ leads to a minimal decrease of σ^*^ but a considerable increase of bias due to a possible background. The presence of the background will eventually influence the choice of *t*_2_, as discussed above. The optimal *t*_1_ is then determined by the Scheme (G1 or G2) and the type of noise, as shown in Table 1. It is minimally dependent on the chosen *t*_2_, with the exception of the G1 scheme with Poisson noise, where it varies between 1 and 1.6 depending on *t*_2_, as can be seen in Figure 4.

## 5 CONCLUSIONS

The presented analysis allowed us to find the optimal experimental parameters *t*_1_ and *t*_2_ for the recently introduced variable‐velocity scanning method for lifetime determination. The optimal parameter values were found to be independent of the signal intensity and were determined by the decay rate *k*. The range of optimal *t*_2_ is relatively broad, with *t*_2_ being sufficiently high so that the conditions are close to the steady‐state regime. The value of *t*_1_ is chosen accordingly, but the precise choice is not critical, as the standard variation of the decay rate is only weakly sensitive to *t*_1_ variation around its minimum (Figure 4). If a broad range of decay rates is measured, as may often be the case in lifetime imaging with a considerable spatial lifetime variation, the values at the lower end of the expected range of *k* (longer lifetimes) should be used to determine the optimal times *t*_1_ and *t*_2_. Importantly, in the scanning scheme, high *t*_2_ assures both minimal standard deviation and minimal bias due to background, simplifying the choice of *t*_1_ and *t*_2_.

Contrary to the scanning Scheme S, compromise has to be sought for the gating Schemes G1 and G2 when considering both standard deviation and bias due to background: while the criterion of minimum standard deviation favors larger *t*_2_, minimizing the effects of background on the bias of *k* calls for smaller *t*_2_.

Furthermore, the sensitivity to the background‐induced bias, quantified by ∣*β*∣, is stronger for the gating Schemes G1, G2 than for S. The higher robustness of the scanning Scheme S compared to the gating schemes constitutes a clear advantage in practical applications.

In this work, we have considered measurements using two scan velocities, which is the minimum required to determine the luminescence lifetime. Employing more than two scan velocities is expected to expand the possibilities of the scanning method, similarly to using more gates in the time‐gating approaches. More scan velocities would mean that a broader range of lifetimes could be optimally measured within one experiment. Furthermore, non‐exponential kinetics could be detected, and more advanced analysis methods analogous to those used with conventional lifetime imaging, either on a pixel‐by‐pixel basis or as global image analysis, could be applied.
